# The Benign Side of the Abdominal Wall: A Pictorial Review of Non-Neoplastic Diseases

**DOI:** 10.3390/diagnostics12123211

**Published:** 2022-12-17

**Authors:** Giorgia Porrello, Federica Vernuccio, Eduardo Alvarez-Hornia Pérez, Giuseppe Brancatelli, Roberto Cannella

**Affiliations:** 1 Department of Biomedicine, Neuroscience and Advanced Diagnosis (Bi.N.D), University of Palermo, Via del Vespro 129, 90127 Palermo, Italy; 2 Institute of Radiology, Department of Medicine-DIMED, University of Padova, 35128 Padova, Italy; 3 Clinica IMQ Zorrotzaurre, 48014 Bilbao, Spain; 4 Department of Health Promotion, Mother and Child Care, Internal Medicine and Medical Specialties (PROMISE), University of Palermo, Via del Vespro 129, 90127 Palermo, Italy

**Keywords:** abdominal wall, incidentalomas, diagnosis, differential, ultrasonography, computed tomography, magnetic resonance imaging

## Abstract

The abdominal wall is the location of a wide spectrum of pathological conditions, from benign to malignant ones. Imaging is often recommended for the evaluation of known palpable abdominal masses. However, abdominal wall pathologies are often incidentally discovered and represent a clinical and diagnostic challenge. Knowledge of the possible etiologies and complications, combined with clinical history and laboratory findings, is crucial for the correct management of these conditions. Specific imaging clues can help the radiologist narrow the differential diagnosis and distinguish between malignant and benign processes. In this pictorial review, we will focus on the non-neoplastic benign masses and processes that can be encountered on the abdominal wall on cross-sectional imaging, with a particular focus on their management. Distinctive sonographic imaging clues, compared with computed tomography (CT) and magnetic resonance (MR) findings will be highlighted, together with clinical and practical tips for reaching the diagnosis and guiding patient management, to provide a complete diagnostic guide for the radiologist.

## 1. Introduction

Benign abdominal wall lesions comprise a wide spectrum of conditions, including benign masses, tumors, and mimickers of neoplastic conditions. Abdominal wall masses generally present as unexpected incidental findings during radiological examinations performed for other clinical reasons, most commonly during computed tomography (CT), and therefore usually represent a clinical dilemma. In many cases, the likely diagnosis can be reached by combining clinical history (i.e., prior therapies, oncologic history, recent surgery, or trauma) and imaging features [1]. However, some lesions still need to undergo a biopsy for the final diagnosis to be made.

Ultrasonography (US) is commonly the first-line exam performed for palpable superficial lesions, providing information on echogenicity, gross vascularization, relationships with nearby vessels, and elasticity. US is also usually used to guide interventional procedures and biopsies. Computed tomography (CT) provides anatomical information on mass extension and involvement of deeper abdominal wall structures, and it is usually preferred in emergency settings or abdominal traumas. Magnetic resonance (MR) is a second-line examination that helps understand the content and nature of a mass and is also useful in selected cases of benign non-neoplastic diseases that require further diagnostic assessment, such as endometriosis [2].

The differential diagnosis among abdominal wall masses can be performed by adopting a clinical–radiologic flowchart. The diagnostic flowchart first includes the differentiation between a mass and a mass-like process. Then, the internal composition of the mass should be determined (e.g., fat, fluid, solid, or fibrous). Then, according to the main components, laboratory results, and clinical history, a differential diagnosis should be made. Based on the origin, abdominal non-neoplastic diseases include infectious processes (e.g., abscesses, necrotizing fasciitis, granulomas, gossypibomas, and fistulae), congenital conditions (e.g., urachal anomalies, defects of the abdominal wall, manifestations of muscular dystrophies, arteriovenous malformations), acquired vascular conditions (e.g., shunts, pseudoaneurysm, and hematomas), and storing and miscellaneous diseases (e.g., injection granulomas from silicone, paraffin, or other drugs; calcinosis; collagen diseases; splenosis; and endometriosis) [1,2,3,4].

This pictorial review aims to depict the spectrum of benign non-neoplastic diseases that may be encountered in the abdominal wall, providing tips for differential diagnosis and their management. For each case, key imaging aspects will be highlighted, drawing a diagnostic guide for the radiologist.

## 2. Infective Pathologies

Infective conditions present as recently appearing lesions accompanied by clinical signs suggestive of inflammation and infections, such as fever, increase in white blood count or C-reactive protein, and redness. Surgical history has an important role, as does the presence of risk factors such as immunocompromised statuses or open wounds [3].

### 2.1. Abscess

Abdominal wall abscesses can manifest as an extension of intra-abdominal processes or as primary infections [4,5,6,7]. Risk factors include surgery, hematomas, diverticulitis, cholecystitis, appendicitis [7], and Crohn’s disease [5].

**Figure 1:**
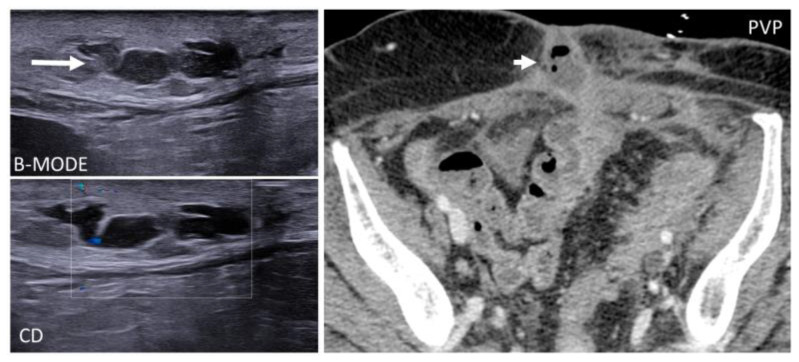
Abscesses on US and CT in a 69-year-old woman with myelofibrosis, presenting with pain along the surgical scar and persistent fever two weeks after splenectomy. B-mode US (first picture, arrow) demonstrates a fluid collection, with no significant vascularization on color Doppler (CD, lower picture). On portal-phase CT (third picture), the abscess is easily distinguishable (arrowhead).

Abdominal wall abscesses present on US as superficial fluid-filled complex lesions, with ill-defined margins, predominantly hypoechoic to surrounding muscles [7,8]. Peripheral hyperemia can be seen on Color Doppler US (CDUS) [3] (Figure 1). To define the extent of the abscess, contrast-enhanced CT and MR could be used in severe cases.

Over time, an abscess becomes walled off by vascularized connective tissue, which corresponds to an enhancing rim, best seen on the venous phase [9]. The enhancing rim surrounds a central low-density necrotic area [6] that contains small air bubbles, suggestive of bacterial degeneration in around 60% of cases [3]. On MR, abscesses show homogeneously high T2 signal in early phases, which later becomes heterogeneous, as the amount of necrosis, gas, and proteinaceous debris increases [9]. Subcutaneous abscesses are important to recognize to avoid systemic evolution and commonly require drainage, along with antibiotics [7]. Percutaneous drainage can be performed by open incision or under ultrasound or CT guidance, according to the location.

### 2.2. Necrotizing Fasciitis

Necrotizing fasciitis (NF) is a soft tissue necrotic infection that involves the fascia and subcutaneous tissue [10]. If not promptly diagnosed, NF may rapidly lead to sepsis and multiple organ failure (MOF) [11], with a mortality of 70–80% [10,11,12,13]. Risk factors include intravenous drug use, diabetes mellitus, immunosuppression, obesity, and peripheral vascular diseases.

**Figure 2:**
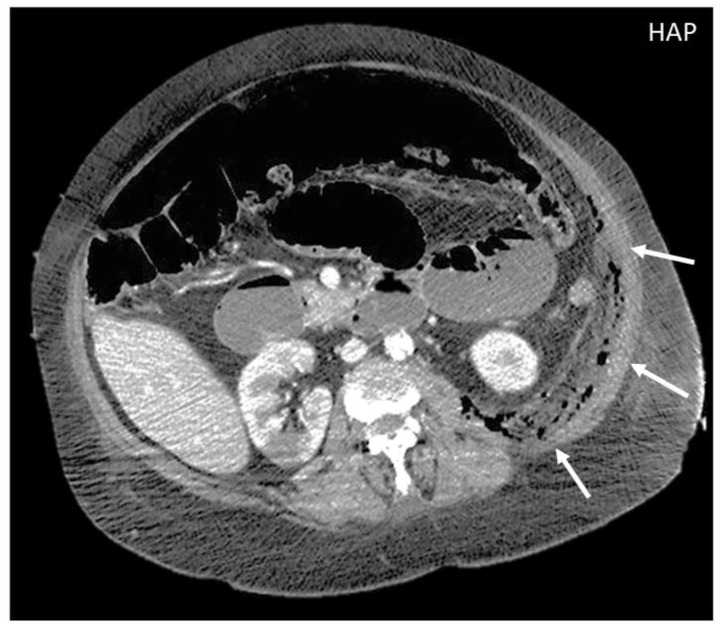
CT appearance of necrotizing fasciitis in a 58-year-old woman with a recent left lower limb open wound who arrived at the ER in septic shock. Arterial phase axial CT scan reveals the presence of free air in the fascial planes of the left lateral and posterior abdominal wall, with fascial thickening and lack of muscular enhancement, as compared to its counterpart (arrows). These elements are suggestive of necrotizing fasciitis. The patient was promptly referred to surgery, but she died on the operatory table.

On CT, the most suggestive findings of NF are the thickening of the fascia and a large amount of subcutaneous gas, although the latter is not specific [10,11,12] (Figure 2).

Focal or diffuse non-enhancing areas [10,11] with extensive multi-compartmental involvement (meaning that at least three muscle compartments are involved in this change) are another typical finding.

On MR, high-signal-intensity areas may be observed on T2-weighted sequences, representing necrosis and edema, alternated with intralesional gas. On T1-weighted sequences, loss of muscle texture and possible high signal intensity compatible with intramuscular hemorrhage might be seen [10,11]. A necrotizing infection mostly requires urgent and aggressive surgical debridement, together with antibiotic therapy and hemodynamic support as necessary.

### 2.3. Foreign Body Retention: Granuloma and Gossypiboma

**Figure 3:**
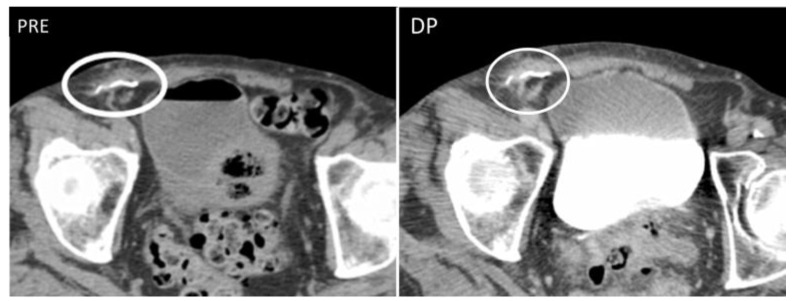
Granuloma occurring along a surgical mesh for an inguinal hernia repair. Axial CT on unenhanced (image on the left) and delayed phase at 10 min (image on the right) shows the formation of a nodular, fibrotic mass, with uptake contrast in delayed phases around a surgical mesh (circle).

Granulomas are protective responses to destroy or sequester particles that are deemed harmful to the body, frequently seen in the context of chronic infections, inflammatory diseases, and foreign bodies. On US, retained foreign bodies are usually hyperechoic with posterior acoustic shadowing, although their appearance depends on their content [3]. As granulomas develop, they appear as well-defined nodular lesions with a peripheral hyperechoic, ill-defined halo that slowly becomes hypoechoic and then hypervascular [3,8]. CT demonstrates a soft tissue lesion with punctuate or gross calcifications, surrounded by fat stranding [5]. Slight peripheral contrast uptake is seen in chronic stages (Figure 3).

Gossypibomas are secondary to the retention of surgical sponges or gauze. The abdominal wall is a rare localization, but this diagnosis should be suspected in post-surgical patients with recurrent infection, fever, and pain. On CT, abdominal wall gossypibomas are thick-walled collections in the abdominal muscles or subcutaneous fat, with an air-like density center and a small fluid component, sometimes with metallic parts [14].

In both conditions, until the foreign body is surgically removed, the inflammation will continue. If unrecognized, they can evolve into systemic infection and septic shock [15].

### 2.4. Fistulae

**Figure 4:**
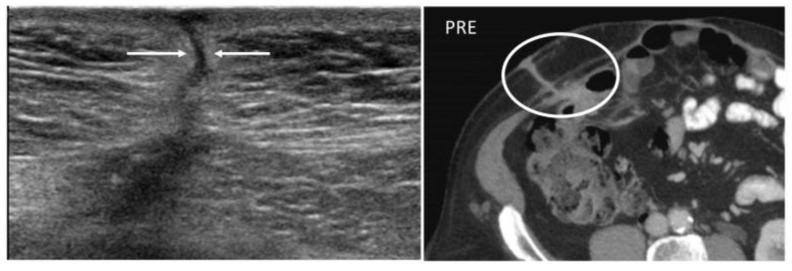
A 22-year-old man with reactivation of Crohn’s disease, presenting as a new enterocutaneous fistula. B-mode US with linear transducer (first picture) shows the presence of a fistulous trait between the skin and a superficial collection (arrows). Axial not-enhanced CT scan (second picture) revealed the course of the fistula, demonstrating communication between the bowel loops and the skin, together with a small collection (circle).

Enterocutaneous abdominal wall fistulae (ECFs) are abnormal connections between a bowel loop and the nearby skin that may be secondary to abdominal surgery or inflammatory bowel diseases. ECFs can either appear collapsed or patent with gaseous or fluid content (“tram-track” appearance) (Figure 4).

Abdominal wall fistulae are seen as hypoechoic or anechoic duct-like structures on US but are better studied on MR or CT, as these latter depict the connection between the bowel loops and the peritoneum or the cutis [16].

Fistulae need to be surgically repaired; otherwise, chronic infections may develop. Abscesses can indeed complicate ECFs: lesions above 2 cm, irregularly large, with thickened walls and patent peripheral sections should raise suspicion for superimposed abscesses [16].

## 3. Congenital Conditions

### 3.1. Urachal Anomalies

Urachal anomalies are due to an incomplete obliteration of the urachus, the embryologic communication between the bladder and the umbilicus [17,18]. According to the location of the patency, four types of anomalies should be distinguished: patent urachus, urachal sinus, urachal cyst, and vesicourethral diverticulum [17].

The differential diagnosis includes malignancies, metastases, and endometriosis. Usually diagnosed during childhood, they are commonly studied on US.

**Figure 5:**
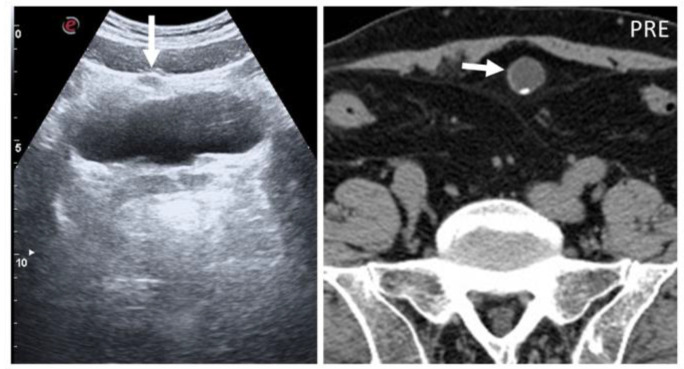
Urachal cysts are found along the superficial planes. On B-mode US, urachal anomalies appear as fluid-filled nodularity close to or in the context of the abdominal wall, incidentally found (arrow). As seen in axial, not-enhanced CT, small parietal calcification can also be seen (short arrow).

In the case of patent urachus, US shows a tubular structure with hypoechoic walls and anechoic content, traced along the anterior abdominal wall [18,19]. Urachal cysts are round midline, homogeneous fluid-filled structures with well-defined walls, located between the bladder and the abdominal muscles, that may contain rim calcifications [17,18,19] (Figure 5).

Umbilical–urachal sinus occurs at the umbilical end of the urachus and is seen as a fusiform outpouching structure into the abdominal wall. Imaging reveals a thickened, fusiform blind dilatation below the navel, with no communication with the bladder. Superinfection can complicate all urachal anomalies, and in that case, peripheral contrast enhancement will be seen [17]. Stone formation can complicate diverticula. Urachal remnants can degenerate into adenocarcinoma, carrying a poor prognosis due to its late presentation. The role of prophylactic surgery in these cases is still highly debated [19].

### 3.2. Congenital Defects of the Abdominal Wall

**Figure 6:**
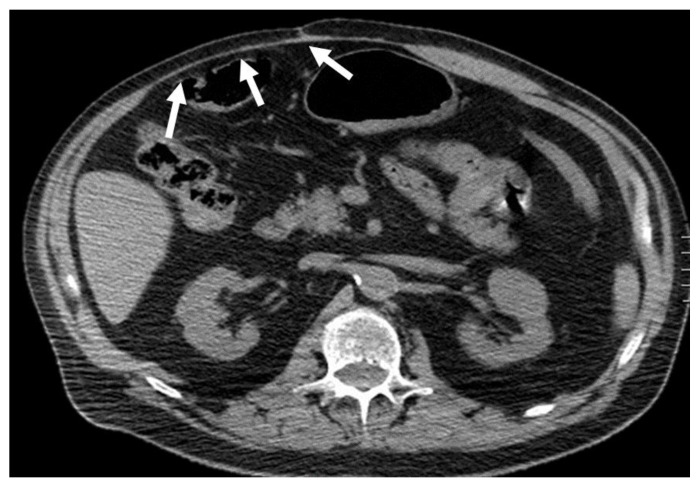
Axial, not-enhanced CT scan shows the congenital absence of the right rectus muscle (arrows).

The most common congenital defect of the abdominal wall is the absence of transversus abdominis muscle, followed by internal oblique and external oblique absence [20], which might be the aftermath of more serious conditions. For example, in infancy, congenital abdominal wall defects can be part of the “prune belly syndrome”, whose main features are abdominal wall flaccidity, urological abnormalities, and cryptorchidism [21] (Figure 6). Radiologists should report the presence of these defects in patients who have to undergo abdominal surgeries because the surgical approach has to be modified accordingly [20].

**Figure 7:**
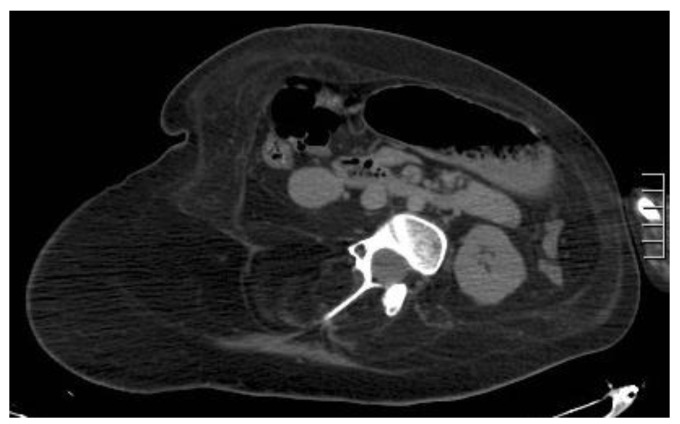
A 23-year-old patient with Duchenne’s syndrome. Axial, not-enhanced CT scan demonstrates complete fat replacement of all abdominal wall muscles with obligated decubitus, as commonly seen in cases of Duchenne’s syndrome patients reaching the ages of 20–30.

Neuromuscular dystrophies can also present defects of the abdominal wall. Duchenne muscular dystrophy is the most common one and is clinically characterized by progressive muscle weakness, starting from the proximal pelvic girdle [22], whose pathognomonic appearance on CT and MR is a progressively extensive fatty replacement of muscles [22,23] (Figure 7).

### 3.3. Arteriovenous Malformations

Arteriovenous malformations (AVMs) are congenital lesions consisting of a tangle of arteries and veins connected by one or more fistulae [8,24].

It is important to distinguish between isolated and syndromic AVMs (e.g., Rendu–Osler disease, PTEN mutations) [25].

AVMs of the abdominal wall are often unilateral, interdigitated with fat, and adjacent to or within the anterolateral muscle groups [13]. Color Doppler US shows focal anechoic vascular dilatations with heterogeneous vascular flow [24]. Contrast-enhanced CT or MRI is performed to depict the relationship with abdominal vessels. Abdominal wall AVMs are usually non-painful superficial lumps. Depending on the site, size, and symptoms, treatment options vary from conservative management to embolization or surgical resection [24].

## 4. Acquired Vascular Conditions

### 4.1. Shunts

Acquired shunts in the abdominal wall might be a sign of portal hypertension or inferior vena cava obstruction, which are both conditions to assess and not underestimate. Indeed, if untreated, the first one may lead to cirrhosis and hepatocellular carcinoma, and the latter can lead to pulmonary embolisms. Acquired shunts are caused by dilatation of the paraumbilical veins within the round ligament [26,27,28].

The paraumbilical veins will connect the superior and inferior epigastric veins in the rectus sheath with the left branch of the portal vein at the umbilicus [29], with an appearance called “caput medusae”.

**Figure 8:**
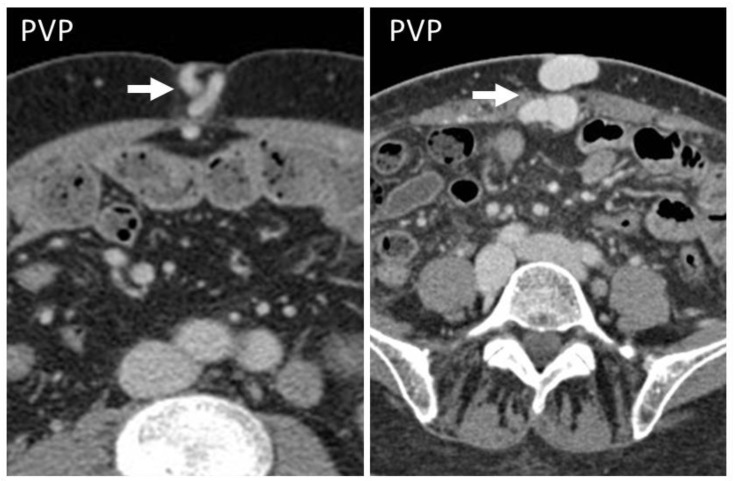
CT appearance of “caput medusae” on axial, contrast-enhanced CT scan on portal venous phase in two cirrhotic patients, as a result of the recanalization of the umbilical vein due to portal hypertension. This appearance is due to tortuous vessels (arrows) reaching the abdominal wall at the level of the navel.

CT should be preferred, in order to study all of the other signs of portal hypertension [27]. Treatment of shunts is linked to the treatment of the underlying causative condition, although in some cases bleeding can also be seen (Figure 8).

### 4.2. Pseudoaneurysm

Pseudoaneurysms, or false aneurysms, originate from the disruption of intimal and medial layers of an arterial vessel, after traumas, infections, or iatrogenic procedures [30,31,32]. On US, pseudoaneurysms are anechoic tubular structures, with thin walls [30,31] and turbulent Doppler flow (“yin–yang” appearance).

**Figure 9:**
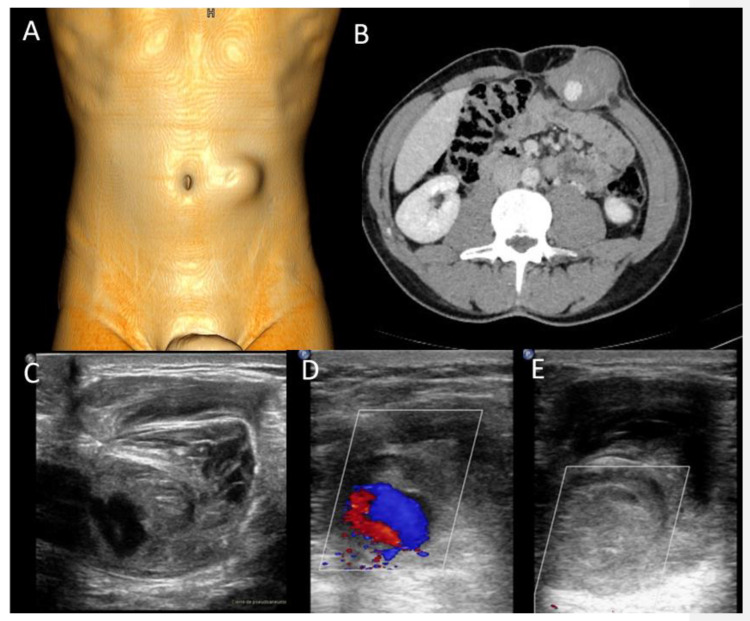
A 37-year-old man presenting with a pulsatile mass with trauma history. Volume rendering cutaneous reconstruction (**A**) and axial, contrast-enhanced portal-phase CT (**B**) showed the presence of a large post-traumatic pseudoaneurysm of the inferior right epigastric artery, with thrombotic apposition. B-mode US and CDUS (**C**–**E**) demonstrate the presence of an anechoic part, corresponding to the contrast-enhanced part on CT, and the heterogeneity of the thrombus. CDUS (**D**,**E**) shows the “yin–yang sign”. The pseudoaneurysm was promptly treated with thrombin injection.

CT angiography is the gold standard for the diagnosis, as it precisely depicts the vascular structures involved [31] and demonstrates the integrity of the pseudoaneurysm sac (Figure 9), which should be smooth unless an infection superimposes.

All pseudoaneurysms should be regarded as surgical urgencies, as they are considered at risk of rupture and therefore should be promptly treated to avoid bleeding [30]. Treatment includes ultrasound-guided thrombin injections and ultrasound compression, endovascular treatments with stent graft placement, and surgical repair. Among minimally invasive procedures, US-guided thrombin injection is usually preferred as it has a higher success rate compared to US-guided simple compression [33].

### 4.3. Hematoma

Abdominal wall hematomas are a common—though often misdiagnosed—cause of acute abdominal pain [34,35]. They mostly involve the rectus muscle (“rectus sheath hematoma”, RSH) and less frequently involve lateral and posterior abdominal muscles [13]. To correctly predict the aftermaths of abdominal wall hematomas, it is important to assess their location. The first step is to locate the arcuate line.

**Figure 10:**
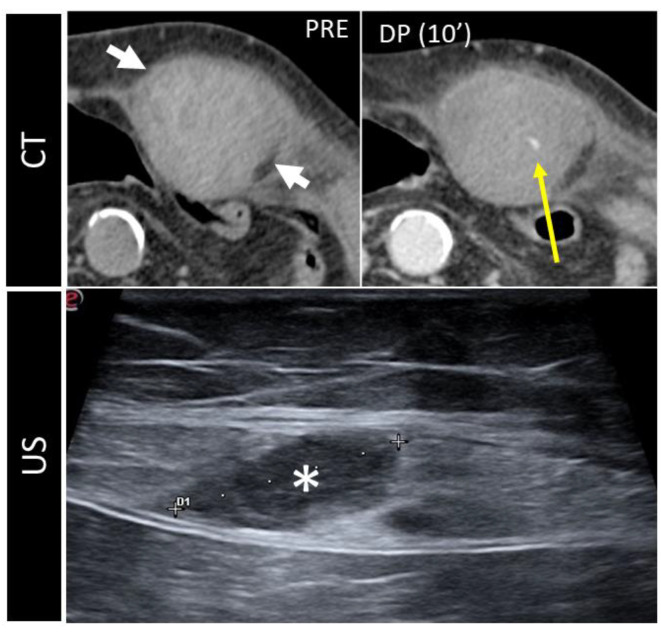
CT and US post-traumatic rectus sheath hematoma above the arcuate line. CT scan shows a well-defined mass, hyperintense on basal scan, in the context of the left rectus sheath (short white arrows). In the second picture, the delayed phase acquired 10 min after the injection of intravenous contrast demonstrates active bleeding (yellow arrow). The third picture shows the same lesion (*), studied some days later on US.

**Figure 11:**
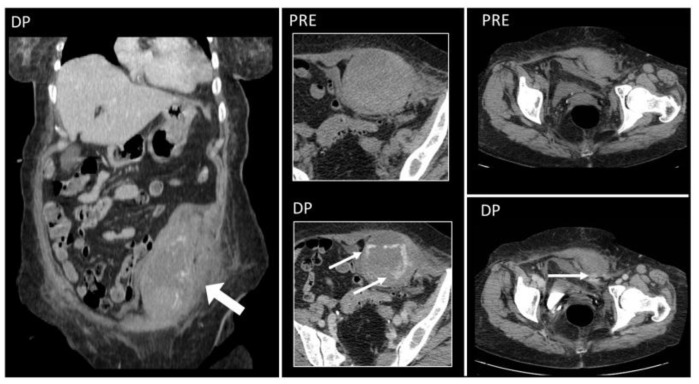
Hematoma below the arcuate line in a 75-year-old woman, with acute myeloid leukemia and low platelet count. After a cough, she started complaining of strong abdominal pain and a mass started growing. Coronal and axial, pre- and post-contrast injection images showed the presence of a large left rectus sheath hematoma (thick arrow) with active arterial bleeding, as demonstrated by the subsequent spreading of contrast in all phases acquired (thin arrows). Since the hematoma was below the arcuate line, bleeding into the prevesical space is also noted (arrow, last picture, bottom row).

Above the arcuate line, RSHs are circumscribed and spindle-shaped (Figure 10); below the arcuate line, there is no anatomical barrier: RSH can cross the midline, and blood can extend into the peritoneum and prevesical space [1,5,13] (Figure 11). 

Hematomas are well-defined masses, with higher attenuation than muscles [9] on unenhanced CT and high-T1, low T2 signals on MR in acute phases [9]. When hematomas are seen, active contrast extravasation must always be ruled out on CT [5,35]. As time passes, the hematoma evolves, and its attenuation becomes lower and lower on CT, reaching serum density after 2–4 weeks [1,5,9]. On chronic stages, the MR signal of hematomas becomes layered. On the periphery, the signal will be low on both T1- and T2-weighted sequences, while on the center, the hematoma will be isointense on T1 sequences and high on T2-weighted ones.

The main consequence of undiagnosed or untreated rectus hematomas is pain and bleeding, which can become life-threatening. Severe bleeding should be promptly identified and aggressively treated with urgent arterial embolization or surgical intervention. Another potential complication is abscess formation, as in any blood collection that is not drained [36].

## 5. Storing and Miscellaneous Diseases

### 5.1. Injection Granulomas

**Figure 12:**
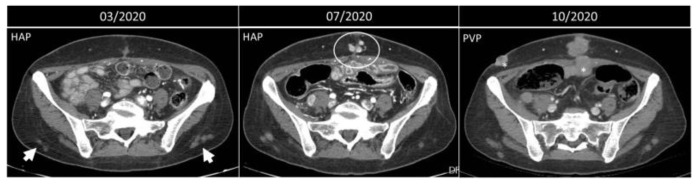
Progression of metastatic disease vs. injection granulomas. Axial, contrast-enhanced arterial and portal-phase CT scans of a 50-year-old woman with NEC of the small bowel. Three months after the surgery, some lesions on the subcutaneous fat of the lower back are noted (arrowheads). Four months later, these nodules are the same size, while along the surgical scar, new enhancing nodules appear (circle). In October 2020, these latter lesions become confluent and bigger, and other lesions appear (*), all compatible with metastatic nodules, while the nodules on the posterior abdominal wall are simple granulomas.

Injection granulomas may develop as an inflammatory response to repeated injections and lead to localized fat necrosis, scar formation, and dystrophic calcification, simulating malignant diffuse processes [9,10,11,12,13] (Figure 12), and require no particular management.

On CT, granulomas are well-defined small nodules with soft-tissue attenuation, often with peripheral calcifications [13]. MR shows T1 hypointense nodules with mild-to-moderate high T2-w signal, depending on the presence of inflammation or fibrosis [9,13].

### 5.2. Silicone and Paraffin

Free silicone and paraffin injections are dangerous beauty techniques, disused but still performed illegally [37]. Serious reactions to silicone injections have been reported, occurring 3 weeks to 23 years after treatment. Aftermaths include strong pain, skin lesions, deformities, recurrent cellulitis and abscesses, granulomas (“siliconoma” and “paraffinoma”) in the subcutaneous and muscular tissue, lymphadenopathy, silicone migration, and necrosis [37,38,39]. Systemic complications have also been reported, including acute pneumonitis, granulomatous hepatitis, organ compression, and sudden death secondary to intravascular embolization [40]. 

Free silicone and paraffin are seen as irregularly scattered collections extended along fascial planes, mimicking sarcomatous or metastatic processes [38,39].

**Figure 13:**
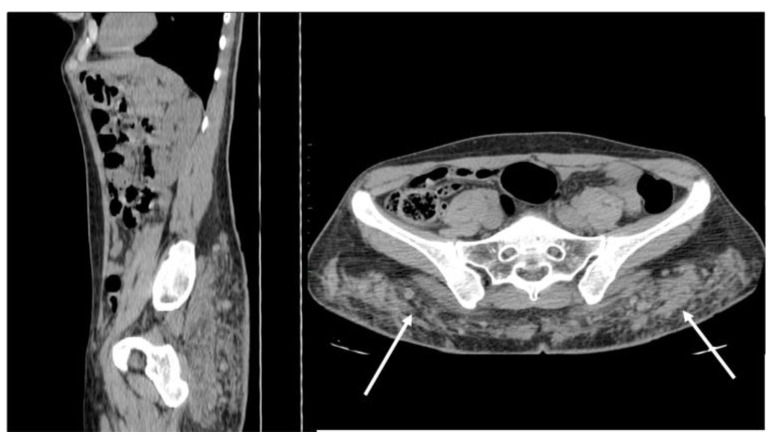
Non-enhanced sagittal and axial CT scan demonstrates free silicon material scattered with an infiltrative appearance, expanding all along the subcutaneous fat of the posterior abdominal wall (arrows). Strand-like lesions coexist with nodular and plaque-like areas, making the differential diagnosis with neoplastic conditions difficult.

On US, they are heterogeneously echoic, with marked posterior acoustic shadowing [39]. On CT, their diffuse infiltrating appearance is well seen, with solid density but a lack of contrast uptake, accompanied by fat stranding [37,38] (Figure 13).

MR appearance comprises a main, plaque-like, ill-defined lesion with a low-to-intermediate signal on T1- and T2-weighted images, but also marked loss of signal on fat-suppression and dual gradient echo sequences in paraffinomas [39]. Even years after the injections, these substances also show high tracer uptake on FDG and gallium PET/CT, making the differential diagnosis with malignancies challenging [38]. Treatment of silicone granulomas is difficult and often unsuccessful. Modalities include topic and systemic corticosteroids, systemic therapies with etanercept or minocycline, liposuction, lasers, and local resection. However, surgical excision is difficult because migration to distant sites results in incomplete removal or wide excisions [40].

### 5.3. Subcutaneous Drugs

**Figure 14:**
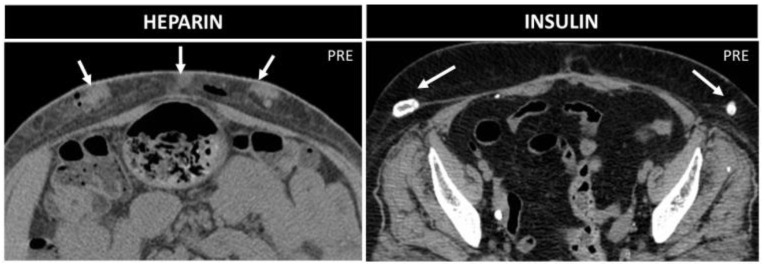
Heparin vs. insulin treatment nodules in two 70-year-old patients. Axial not-enhanced CT in the first patient shows some collections with parenchymatous density, which indicate bleeding, and small air bubbles. In the second patient, instead, lipodystrophies with peripheral calcifications are seen.

Lipohypertrophy is a classical sequela of subcutaneous insulin injections. On CT and MR, it is seen as focal subcutaneous soft-tissue masses, with fat proliferation in a symmetric fashion [41] and calcifications [41,42,43]. “Insulin balls” can also occur, subcutaneous amyloid deposits, visible as soft-tissue masses, with necrotic borders, due to amyloid toxicity [43] (Figure 14).

Management is different between these two conditions: while lipohypertrophy disappears with the suspension of insulin therapy, insulin balls tend to progressively enlarge and require surgical excision [42]. Repeated heparin injections can be associated with air and fluid levels [9]; small soft-tissue nodules, connected to the destruction of the hypodermic fat; and small hematomas with adjacent hazy soft tissue [44] (Figure 14). There is no special management, only a recommendation to alternate the injection spot when performing heparin injection.

### 5.4. Calcinosis and Collagen Diseases

Systemic alterations in calcium metabolism can lead to extensive subcutaneous calcinosis [13], either due to neoplastic (e.g., paraneoplastic syndromes, metastases) or non-neoplastic conditions (e.g., chronic kidney failure, dystrophic calcifications, gout, granulomas, collagen diseases) [44].

**Figure 15:**
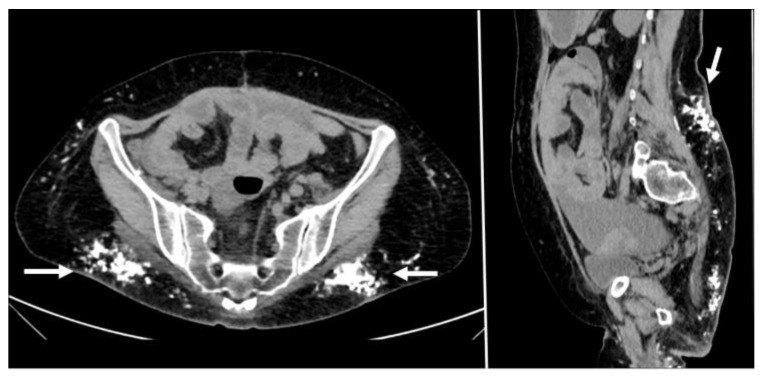
Woman with long-standing dermatomyositis. Axial and sagittal not-enhanced CT scan reveals diffuse, multiple calcifications on the subcutaneous abdominal tissue, representing the evolution of non-controlled dermatomyositis. Large peritoneal effusion is also seen.

Calcinosis Universalis is the main subtype seen in collagen vascular diseases and is recognized on CT as sheet-like deposits of calcifications in the subcutaneous tissue and fascial planes, mostly with intramuscular distribution [13,45] (Figure 15).

Currently, we lack specific guidelines for managing calcinosis cutis in autoimmune connective tissue disorders. An intensification of immunosuppressive therapy was suggested, but no specific treatment was recommended. Rituximab and bisphosphonates have been increasingly used with success in dermatomyositis skin lesions [45]. Surgery, laser, and physical therapies should be considered in extensive calcinosis. However, surgical management can lead to skin necrosis and a limited range of motion [45].

### 5.5. Splenosis

Splenosis is the heterotopic auto-transplantation of splenic tissue that occurs after a traumatic or surgical rupture of the spleen; it is usually asymptomatic and without any degeneration risk [46]. The abdominal wall is a rare location that may resemble subcutaneous or intramuscular tumors [47,48]; enlarged lymph nodes, hernias, and endometriosis [46]; or peritoneal carcinomatosis [47,48,49].

**Figure 16:**
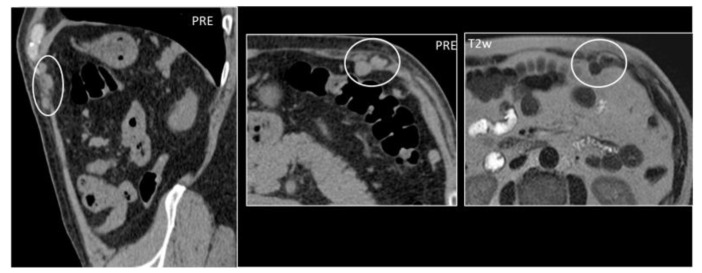
A 59-year-old woman who underwent splenectomy some years prior. During a CT scan, new, rounded masses (circles) were found along the peritoneum and the left rectus abdominis muscle. These formations show parenchymal attenuation on CT (pictures 1 and 2) and share the same T2 intensity as the spleen. These lesions were later characterized as splenosis.

US appearance is non-specific as it shows round or oval-shaped, hypo- to isoechoic solid masses with posterior acoustic enhancement and an incomplete hyperechoic rim [47]. CT and MR will show round lesions with imaging and contrast behavior similar to the spleen. In doubtful cases, heat-denatured blood cell scintigraphy should be considered [46,47] (Figure 16). Splenosis does not require follow-up or pose a risk for degeneration. Evidence shows that there is no need for surgical excision since it represents normal-functioning splenic tissue [47].

### 5.6. Endometriosis

Abdominal wall endometriosis (AWE) manifests as solid nodules, with occasional cystic changes due to intralesional bleeding [50]. AWE originates from the embedding of endometrial tissue in the abdominal wall, which develops ventrally and infiltrates the rectus or oblique muscles, appearing as nodular masses known as endometriomas [50,51,52]. The differential diagnosis includes abscess, lipoma, hematoma, sebaceous cysts, granuloma, sarcomas, and desmoid tumors [9]. Consequences vary according to the location, ranging from muscular or nearby organ damage to bleeding.

**Figure 17:**
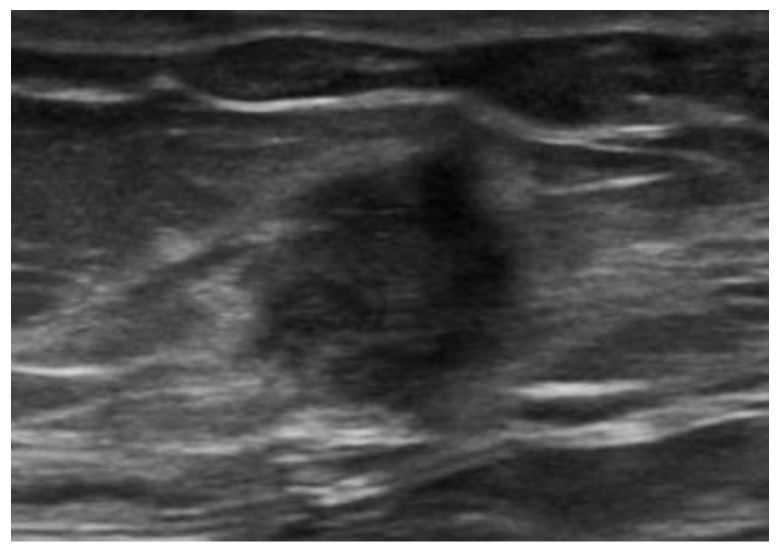
Endometrioma of the abdominal wall on US: heterogeneously hypoechoic, round, or oval-shaped nodule, with scattered internal echoes.

US demonstrates heterogeneously hypoechoic, round or oval-shaped nodules or masses, with scattered internal echoes, and small cystic areas [9,50,53,54], surrounded by a hyperechoic rim [55] (Figure 17).

**Figure 18:**
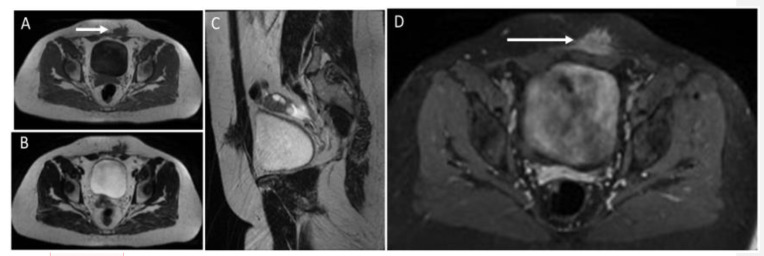
MR study of an endometriotic nodule (arrows) in the left rectus abdominis muscles and subcutaneous tissue. Endometriotic implants inside muscle are well characterized on MR. They show heterogeneously low signal on T1 (**A**) and T2 images (images **B**,**C**) and strong enhancement after the injection of contrast agent (**D**). Sagittal reconstruction (**C**) shows the extension of the endometriotic nodule into fat tissue, fascia, and intramuscular location.

CT shows solid masses with ill-defined borders, isoattenuating to the surrounding muscles [55]. After contrast injection, endometriosis may show slight enhancement [50,53,54] and a characteristic “Gorgon sign”, corresponding to linear strands irradiating peripherally from the central nodules [55]. MRI provides excellent contrast resolution and demonstrates the depth of infiltration, gives better tissue definition, and shows the integrity of the surrounding muscle tissue [54]. On MR, abdominal endometriosis is hyperintense on T2-weighted images [50,54,55]; isointense to muscles on T1-weighted sequences, with foci of high signal, suggestive of hemorrhage [50,56]; and presents high signal lesions on fat-saturated T2-weighted images, with moderate enhancement [54,56] (Figure 18). In addition, diffusion-weighted imaging (DWI) may be useful for differentiating AWE from tumors. Regarding endometrial cysts, AWE tends to have lower apparent diffusion coefficient (ADC) values compared with other cysts, due to blood content [50]. However, compared to pelvic endometriosis, while the latter varies from purely cystic chocolate cysts to solid deposits or fibrosis, AWE will show almost invariably a pure solid aspect.

The clinical presentation is heterogeneous. No particular constellation of clinical risk factors has been identified, and the histological report is the major diagnostic tool for confirmation. Imaging including ultrasound and magnetic resonance imaging can assist with the localization of the lesions and aid in surgical excision, which is the first line of therapy [57]. AWE lesions that have been removed in their entirety are unlikely to reoccur [58].

### 5.7. Abdominal Wall Adhesions

Adhesions at the level of the abdominal wall after surgery may occur in about 39% of patients at risk for adhesions [59]. Indeed, abdominal entry at the time of surgery (e.g., laparoscopy, laparotomy) is a critical step with a risk of injury to underlying viscera owing to bowel adhesions. As the most common laparoscopic point of entry is the umbilical site, many studies investigated abdominal wall adhesions at the periumbilical site demonstrating an approximate bowel adhesion rate of 12% at this level [59]. US allows the assessment of adhesion by investigating the slide of viscera underneath the abdominal wall, with a sensitivity of 91% and specificity of 93% for any site and a sensitivity of 96% and specificity of 93% for the periumbilical site [59]. Abdominal wall adhesions can be more accurately assessed through MRI [60]. MRI studies using cine-MRI to identify abdominal wall adhesions are conducted by running several imaging cycles while instructing the patients to use Valsalva’s maneuver to increase their intra-abdominal pressure and thus provoke the movement of abdominal contents in relation to each other. The lack of visceral slide and separation between organs as well as between organs and the abdominal wall may be interpreted as a sign of adhesion [61]. However, MRI tends to over-diagnose adhesions [60]. The clinical presentation is heterogeneous, from asymptomatic to abdominal distension, pain, nausea, and abnormal bowel movement pattern. The clinical benefit of identification of abdominal wall adhesion after surgery is two-fold: first, patients with abdominal wall adhesions are at increased risk for bowel and mesenteric injury following blunt abdominal trauma and for developing small bowel obstruction; second, knowledge of the location of abdominal wall adhesions allows choosing alternative abdominal entry points for any eventual new surgery in that patient [62,63]. Lysis of abdominal wall adhesions is considered among the therapeutic options [64].

## 6. Conclusions

The spectrum of benign non-neoplastic abdominal wall diseases is wide. Some benign conditions are easily diagnosed and, in most cases, do not require any further diagnostic workup or follow-up. However, some cases can be challenging, may require further diagnostic assessment, and may need proper urgent treatment or follow-up. Radiologists must be aware of the key imaging features that allow a prompt differential diagnosis among these different diseases, to avoid unnecessary follow-up or overtreatment on one hand and to avoid misdiagnosis or delayed treatment on the other hand. Even though in some cases histology will still be needed to reach the final diagnosis, the role of the radiologist is fundamental to curtail the differential diagnosis and correctly interpret these processes non-invasively.

## Data Availability

Not applicable.
